# Complementary Use of Super-Resolution Imaging Modalities to Study the Nanoscale Architecture of Inhibitory Synapses

**DOI:** 10.3389/fnsyn.2022.852227

**Published:** 2022-04-08

**Authors:** Sara E. Gookin, Matthew R. Taylor, Samantha L. Schwartz, Matthew J. Kennedy, Mark L. Dell’Acqua, Kevin C. Crosby, Katharine R. Smith

**Affiliations:** Department of Pharmacology, University of Colorado School of Medicine, Aurora, CO, United States

**Keywords:** super-resolution microscopy, 3D-SIM, *d*STORM, inhibitory synapse, neuroligin-2, gephyrin

## Abstract

The nanoscale architecture of synapses has been investigated using multiple super-resolution methods, revealing a common modular structure for scaffolds, neurotransmitter receptors, and presynaptic proteins. This fundamental organization of proteins into subsynaptic domains (SSDs) is thought to be important for synaptic function and plasticity and common to many types of synapses. Using 3D super-resolution Structured Illumination Microscopy (3D-SIM), we recently showed that GABAergic inhibitory synapses exhibit this nanoscale organizational principle and are composed of SSDs of GABA_*A*_ receptors (GABA_*A*_Rs), the inhibitory scaffold gephyrin, and the presynaptic active zone protein, RIM. Here, we have investigated the use of 3D-SIM and *d*STORM to analyze the nanoscale architecture of the inhibitory synaptic adhesion molecule, neuroligin-2 (NL2). NL2 is a crucial mediator of inhibitory synapse formation and organization, associating with both GABA_*A*_Rs and gephyrin. However, the nanoscale sub-synaptic distribution NL2 remains unknown. We found that 3D-SIM and *d*STORM provide complementary information regarding the distribution of NL2 at the inhibitory synapse, with NL2 forming nanoscale structures that have many similarities to gephyrin nanoscale architecture.

## Introduction

In recent years neuronal synapses have been studied using a range of super-resolution modalities (for a recent review, see Werner et al., 2021). A key finding from this body of work is that synaptic scaffolds, neurotransmitter receptors, and adhesion molecules are not distributed uniformly at synaptic sites, but are often clustered into regions of high density within the synapse, termed nanodomains or subsynaptic domains (SSDs; Biederer et al., 2017; Yang and Specht, 2019). This organization appears to be a common feature of multiple different types of synapses and thought to be important for function and plasticity (Broadhead et al., 2016; Tang et al., 2016, Hruska et al., 2018; Crosby et al., 2019, Ramsey et al., 2021). Our recent work demonstrates that GABAergic inhibitory synapses, which mediate synaptic inhibition in the central nervous system, also exhibit this nanoscale organizational principle (Crosby et al., 2019; Garcia et al., 2021). Through the use of super-resolution three-dimensional structured illumination microscopy (3D-SIM) we found that synaptic GABA_*A*_Rs, the inhibitory synaptic scaffold gephyrin, and the presynaptic active zone protein, RIM, all form SSDs. These structures associate with each other and emerge in synapses as it they grow during plasticity, suggesting that this modular structure might underlie activity-dependent synaptic plasticity (Crosby et al., 2019).

Thus far, we have delineated some key elements of inhibitory synaptic architecture with 3D-SIM. However, we reasoned that complementing this approach with a fundamentally distinct super-resolution method, direct stochastic optical reconstruction microscopy (*d*STORM), would provide further information about the synapse and its organization at different spatial scales, allowing us to compare the information provided by these two powerful techniques. 3D-SIM and *d*STORM derive a super-resolved image in distinct ways. 3D-SIM uses a series of raw images acquired with varied patterned illumination orientations that are processed in the Fourier domain, allowing access to higher-frequencies. Upon reverse transform, this higher frequency information corresponds to finer spatial resolution, ∼120 nm laterally and ∼300 nm axially, in the resulting image reconstruction (Gustafsson et al., 2008). In comparison, the raw data in *d*STORM is obtained by imaging a densely labeled sample over several thousand frames. In each individual frame only a small proportion of dye molecules emit photons, detected as “blinks” sparsely spread across the field. This spatial separation allows for fitting the point-spread function from each of these emission events to a precision on the order of 10–20 nm laterally. When the localizations from all the single-frames are aggregated, a pointillistic super-resolution representation of the labeled target emerges (van de Linde et al., 2011).

To investigate the complementary use of 3D-SIM and *d*STORM in the analysis of inhibitory synapses, we chose to image the inhibitory synaptic adhesion molecule, neuroligin-2 (NL2). NL2 is a crucial mediator of inhibitory synapse formation and organization (Oh and Smith, 2019), and interacts with gephyrin and GABA_*A*_Rs (Poulopoulos et al., 2009; Yamasaki et al., 2017). This association with the key players of the inhibitory synapse suggests that NL2 could contribute to the nanoscale organization of GABA_*A*_Rs and gephyrin, yet its nanoscale sub-synaptic distribution has not been described. By imaging gephyrin and NL2 with both approaches, we find that NL2 forms nanoscale structures at inhibitory synapses. 3D-SIM and *d*STORM provided complementary information and overlapping structural information about NL2 distribution, with *d*STORM providing a more detailed landscape for NL2 architecture. However, despite the difference in spatial resolution, both approaches provided similar biological conclusions, supporting the use of multiple super-resolution techniques to decipher the nanoscale architecture of synapses.

## Materials and Methods

### Dissociated Hippocampal Cultures

Animal procedures were conducted in accordance with the National Institutes of Health (NIH) *Guide for the Care and Use of Laboratory Animals* and approved by the Institutional Animal Care and Use Committee at the University of Colorado. Rat primary hippocampal neurons were cultured and dissociated in papain from mixed sex postnatal day 0–1 rats as previously described (Crosby et al., 2019; Rajgor et al., 2020). The neurons were seeded on 18 mm glass #1.5 coverslips coated with poly-D-lysine at a density of 150,000–200,000 cells in MEM containing 10% FBS and Penicillin-Streptomycin. Media was replaced 24 h post seeding with Neurobasal-A Medium supplemented with B-27 and GlutaMAX. Cells were grown for 15–18 days at 37°C, 5% CO2 and fed every 5 days. Mitotic inhibitors (uridine fluorodeoxyuridine) were added at day 5 to limit growth of actively dividing cells.

### Immunocytochemistry

For 3D-SIM and *d*STORM, neurons were fixed in 4% PFA solution [4% sucrose, 1× PBS and 50 mM HEPES (pH7.5)] for 5 min at room temperature followed by three washes with 1× PBS. Neurons were blocked for 1 h (5% BSA, 2% Normal Goat Serum, 0.5% NP-40 and 1× PBS) and incubated with antibodies to Gephyrin mAb7a (1:500 Synaptic Systems 147 011), VGAT (1:1000 Synaptic Systems 131 004) and NL2 (1:500 Synaptic Systems 129 203) in block for 1 h. Neurons were washed three times in 1× PBS and then labeled with secondary antibodies for 1 h at room temperature (for 3D-SIM; 1:1000 ThermoFisher Alexa Fluor 488, 568 and 647), or overnight (for *d*STORM; 1:1000 Alexa Fluor 647 anti-mouse; 1:1000 Sigma-Aldrich CF568 anti-rabbit; 1:1000 ThermoFisher Alexa Fluor 488 anti-guinea pig). Coverslips were washed four times in 1× PBS and mounted on glass microscope slides using ProLong Gold Antifade mounting media for 3D-SIM. For *d*STORM, coverslips were washed and fixed with 4% PFA in 1× PBS for 5 min at room temperature followed by final washes where they remained in 1× PBS until imaging.

### 3D Structured Illumination Microscopy

Images were acquired with a Nikon SIM-E Structured Illumination super-resolution microscope equipped with a 100×, 1.49 NA objective; an ORCA-Flash 4.0 sCMOS camera (Hamamatsu); and Nikon Elements software. To maximize signal to noise and reduce photobleaching, acquisition conditions and camera integration time were set in a similar manner to Crosby et al. (2019). On average, 8–15 ROIs (individual synapses) were manually selected per neuron (4–5 neurons) for each condition, with a biological replicate of 3 neuronal cultures for a total of 130–180 synapses (12–15 neurons) per condition. Synapses were within the entire Z-stack, and each selection was based off VGAT positive staining. A high throughput pipeline for analysis is as follows: synapses were processed by background subtraction (ImageJ), image segmentation (split-Bregman/MOSAIC suite) (Rizk et al., 2014), and geometric analysis (MATLAB) as further detailed in Crosby et al. (2019). For image segmentation, the following parameters were utilized: ‘‘Subpixel segmentation,’’ ‘‘Exclude Z edge,’’ Local intensity estimation ‘‘Medium,’’ Noise Model ‘‘Gauss.’’ All 3D-SIM imaging analysis was performed blind to experimental condition. 3D rendering of SIM data was achieved using the function CaptureFigVid.m.^1^

### *d*STORM

Samples were imaged in a standard *d*STORM buffer containing 50 mM Cysteamine hydrochloride, 10% glucose, 0.6 mg/mL Glucose Oxidase from Aspergillus niger, 0.063 mg/mL Catalase from Bovine liver in PBS, pH between 7.5–8.0. Imaging was performed on a Zeiss Elyra P.1 TIRF microscope using a Zeiss alpha Plan Apochromat TIRF 100×/1.46 NA oil objective (Zeiss Item # 420792-9800-720) and a tube lens providing an extra factor of 1.6× magnification. Alexa647 and CF568 dyes were imaged in sequential time-series of approximately 20,000 frames each. Image size was 256 × 256 pixels, integration time was 18 ms for both channels. Alexa-647 molecules were ground-state depleted and imaged with a 100 mW 642 laser at 100% AOTF transmission in ultra-high power mode (condensed field of illumination), corresponding to approximately 1.4 W/cm^2^. Emission light passed through a LP 655 filter. CF-568 molecules were ground-state depleted and imaged with a 200 mW 561 laser at 100% AOTF transmission in ultra-high power mode, corresponding to approximately 2.5 W/cm^2^. Emission light was passed through a BP 570-650 + LP 750 filter. For each dye, ground-state return was elicited by continuous illumination with a 50 mW 405 laser at 0.01–0.1% AOTF transmission. Excitation light was filtered by a 405/488/561/642 filter placed in front of the camera. Images were recorded with an Andor iXon + 897 EMCCD. The camera EM gain was set to 100, which yields an effective conversion of 1 photo electron into 1.65 digital units. The image pixel size was 100 nm *xy*.

### Processing

Raw data was processed through a custom written pipeline written in MATLAB (Mathworks) made up of a number of modular elements, described briefly. The Bio-Formats MATLAB toolbox (Linkert et al., 2010) was used to read Zeiss raw data files into MATLAB. Image data was transferred between MATLAB and FIJI using MIJI.^2^ If necessary, raw data was pre-processed with a temporal filter (Hoogendoorn et al., 2014) to remove non-homogeneous background. The filter radius was set at 51 frames, with a key frame distance of 10 (filter is explicitly calculated only for every 10 frames and interpolated between), the quantile for the filtering was set a 20%. Localization of dye emitters was performed using the ThunderSTORM ImageJ plugin (Ovesny et al., 2014). The camera EM gain was set to 100, which resulted in a photon-to-ADU of 1.65. When the temporal median filter was used, the Offset was set to zero. Image filtering was done with the Wavelet filter setting, with a B-Spline order of 3 and scale of 2.0. A first pass approximate localization of molecules was achieved with by finding local maximum with a peak intensity threshold of 3*std(Wave.F1) and 8-neighborhood connectivity. Weighted least squares fitting of the PSF to achieve sub-pixel localizations was achieved by use of an integrated Gaussian with a fitting radius of four pixels and an initial sigma of 1.2. Localizations were filtered based on the attributes of uncertainty (<20 nm), sigma (50–150 nm), and intensity (<10,000 for CF568 and < 15,000 for Alx647). Localizations within 50 nm were merged with a frame-gap allowance of 1.

Before each experiment a calibration was calculated to correct for shifts and distortions between the acquired fluorescent channels. Sub-diffraction size beads, labeled with fluorophores in both channels were imaged. The bead positions were fitted and registered between the fluorescent channels. Registered localizations from multiple bead images were compiled into one data-set. Calibration matrices of the shift in *x* and *y* direction between the imaging channels across the full field of view were calculated by either applying a 2D polynomial fit or a localized weighted averaging to the registered bead localizations. In the raw data, the shift and distortion between the imaging channels was up to 100 nm. Applying the calibration to the STORM data yields an RMS error of less than 15 nm for the channel misalignment. Drift correction was performed using the redundant cross-correlation method described in Wang et al. (2014). The segmentation parameter was set at 500 frames, the bin size used in the cross-correlation was 10 nm, and the error threshold for the recalculation of the drift was five pixels.

Localizations were rendered into images using the ThunderSTORM visualization module using the method of average shifted histograms with a magnification of 10 and lateral shift of 2 nm. For SIM like renderings, the Normalized Gaussian method was used with a magnification of 2.5 and the lateral uncertainty locked at 60 nm.

### Analysis

Coordinate analysis of our dSTORM data is conceptually similar to methods previously used to classify nanoscale organization at the excitatory synapse (Tang et al., 2016). Synapses for downstream analysis were selected manually from a composite rendered image and ROI coordinates were recorded using a custom ImageJ macro. ROI details were imported into MATLAB using the ReadImageJROI function.^3^ The gephyrin scaffold and NL2 localizations were segmented using a coordinate-by-coordinate density calculation. Briefly, because labeling density could vary greatly, the thresholding parameter was determined from the overall density range of the ROI. Localizations with a local-density in the lower 10% of that range were considered to be outside of the synaptic region/clusters. Boundaries for these regions were delineated using MATLAB’s alphaShape function, with an α value of 100. Only gephyrin regions with an area of 1.5 e3 nm^2^ or greater were considered for analysis. High-density regions (HDRs) were defined by a cutoff determined by randomizing the experimental localizations assuming a uniform distribution across the synaptic region. The local density threshold for an experimental coordinate to be considered as part of a HDR was set at the mean local density of the randomized dataset plus 2 standard deviations. The geometric boundaries of individual HDRs were again delineated using MATLAB’s alphaShape function, with an α value of 7. NL2 HDRs were classified as overlapping with the gephyrin scaffold (or gephyrin HDR) if the overlap area had a fraction of 0.23 or greater of the total NL2 HDR area.^4^

## Results

### Neuroligin-2 Exhibits Non-uniform, Sub-Synaptic Organization at Inhibitory Synapses

**FIGURE 1:**
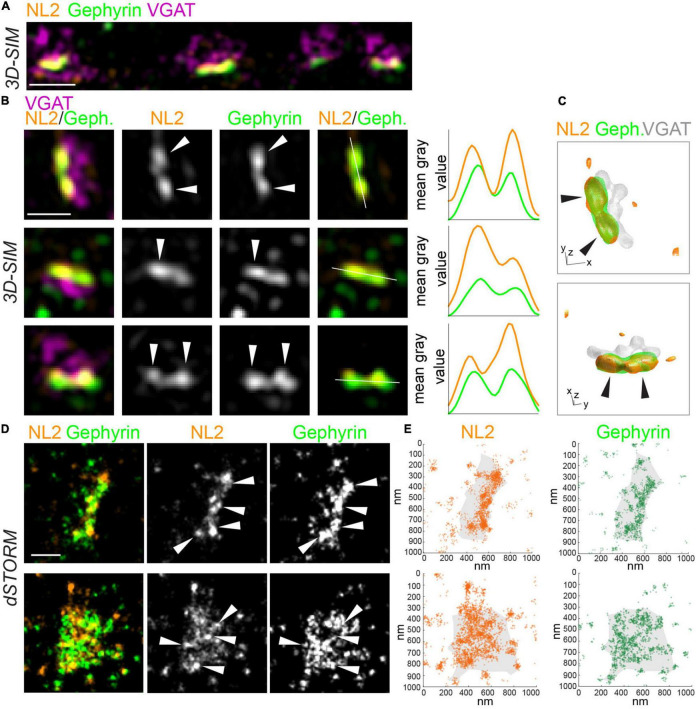
SIM and *d*STORM reveal NL2 is organized into nanoscale structures at inhibitory synapses. **(A)** 3D-SIM maximum projection of hippocampal dendrite labeled with antibodies to NL2, gephyrin and VGAT. Scale bar = 1 μm. **(B)** Magnifications of synapses from 3D-SIM images, with line-scans showing nanoscale subsynaptic domains (SSDs) of NL2 and gephyrin. White lines show path of line scans on the right. Arrows indicate NL2 and gephyrin SSDs. Scale bar = 500 nm. **(C)** 3D reconstruction of inhibitory synapse in panel **(B)**, upper panel, labeled for NL2, gephyrin and VGAT. XY scale bar = 320 nm, Z scale bar = 600 nm. Also see Supplementary Movie 1. **(D)** *d*STORM rendered images of VGAT-positive inhibitory synapses labeled with antibodies to NL2 and gephyrin. Arrows indicate high-density regions. Scale bar = 250 nm. **(E)** Localization plots of *d*STORM data from panel **(D)**.

To characterize the nanoscale architecture of NL2, we imaged NL2 along with the scaffold protein, gephyrin, at inhibitory synapses with both 3D-SIM and *d*STORM. In addition, vesicular GABA transporter (VGAT) was also labeled to mark GABAergic presynaptic terminals (Figures 1A,B). 3D-SIM images revealed that NL2 is often non-uniformly organized into small, nanoscale SSDs, similar to those we have observed for gephyrin and synaptic GABA_*A*_Rs (Figures 1A,B; Crosby et al., 2019; Garcia et al., 2021]. Line-scans through individual synapses supported this observation and indicated multiple peaks of intensity for NL2 and gephyrin within a single synapse (Figure 1B). Furthermore, 3D rendering of the 3D-SIM data underscored the modular nature of NL2 distribution at the synapse (Figure 1C and Supplementary Movie 1). *d*STORM of NL2 and gephyrin also showed a similar modular distribution for NL2 and gephyrin (Figures 1D,E). Both the rendered images and coordinate maps (Figures 1D,E) from our *d*STORM data confirmed the heterogeneous distribution of NL2 and gephyrin across the inhibitory synapse.

### Nanoscale Parameters of Neuroligin-2 Nanostructures Determined by SIM and *d*STORM

**FIGURE 2:**
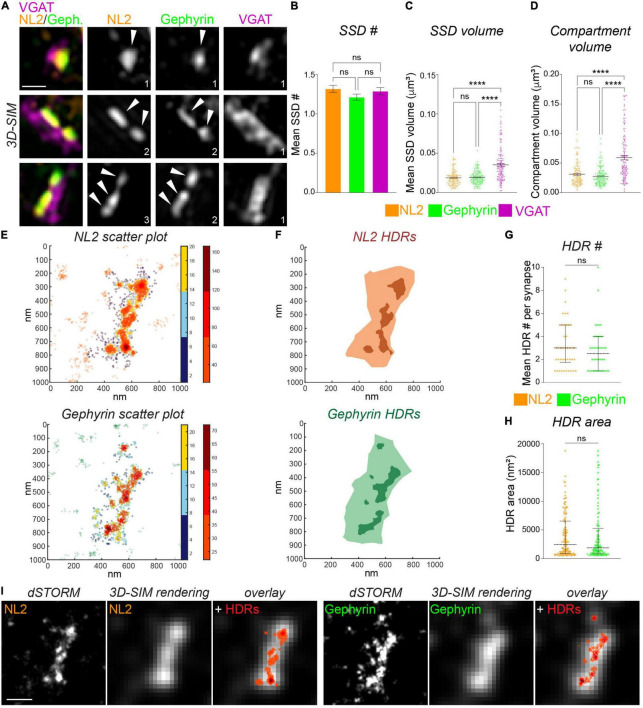
Measurement of NL2 substructure parameters by 3D-SIM and *d*STORM. **(A)** Maximum projection 3D-SIM images of hippocampal inhibitory synapses labeled for NL2, gephyrin and VGAT. Numbers denote number of SSDs in compartment. **(B)** Mean number of SSDs per compartment. *p* = 0.186 (Kruskal–Wallis); *n* = 132 synapses. **(C)** Individual SSD volumes for NL2, gephyrin, and VGAT. ****p < 0.0001 (Kruskal–Wallis, Dunn’s *post hoc*); *n* = 132 synapses. Horizontal line denotes mean. **(D)** Compartment volumes for NL2, gephyrin, and VGAT. ****p < 0.0001 (Kruskal–Wallis, Dunn’s *post hoc*); *n* = 132 synapses. Horizontal line denotes mean. **(E)** Heat maps of NL2 and gephyrin localizations generated by *d*STORM. Hot colors (color-bar 2) represent localizations that are classified as high-density (HDRs), remaining synaptic localizations are shown in cooler colors (color-bar 1). **(F)** Diagrams showing the delineation of NL2 and Gephyrin HDRs (darker shades) and NL2 and gephyrin post-synaptic regions (lighter shades). **(G)** Number of HDRs per compartment for NL2 and gephyrin. Horizontal lines denote median and 25–75% quantiles. *p* = 0.333 (Mann Whitney), *n* = 42 synapses. **(H)** Individual NL2 and gephyrin HDR areas. Horizontal line denotes median and 25–75% quantiles. *p* = 0.518 (Mann Whitney), *n* = 42 synapses. **(I)** Images showing *d*STORM rendered image at *d*STORM and 3D-SIM resolution, with identified HDRs overlaid in red. Scale bar = 250 nm.

Following the imaging of NL2 and gephyrin with 3D-SIM and *d*STORM, we used distinct computational methods to identify and quantify the dimensions of NL2 subsynaptic structures. To identify NL2 SSDs from our 3D-SIM images we utilized our previously developed model-based object 3D-segmentation analysis (Crosby et al., 2019). The number of NL2 SSDs within a synapse varied greatly over the synapses analyzed, with some being formed of a single SSD, whereas others harboring multiple SSDs (Figure 2A). The mean number of NL2 SSDs per synapse was similar to that of gephyrin and VGAT (Figure 2B). Our analysis also showed that NL2 SSDs are of a similar volume to those of gephyrin, but were substantially smaller than VGAT substructures in the presynaptic terminal (Figure 2C). Furthermore, NL2 compartment volumes were also similar to those of gephyrin, but significantly smaller than that of VGAT (Figure 2D), which was in agreement with our previous work (Crosby et al., 2019; Garcia et al., 2021) and the presence of NL2 within the inhibitory post-synaptic domain.

We characterized the finer scale distribution of NL2 and gephyrin captured by *d*STORM by quantifying the local density of the NL2 and gephyrin localizations (see section “Materials and Methods”). Both NL2 and gephyrin exhibited regions of relatively high-density (Figure 2E). We were able to delineate the boundaries of these high-density regions (HDRs) and enumerate their geometric attributes (Figure 2F). The number of HDRs per synapse varied across synapses for both NL2 and gephyrin, but both exhibited ∼3 HDRs per synapse (Figure 2G). The HDR areas for NL2 and gephyrin were also similar (Figure 2H). Despite the 3D/2D disparity between our SIM and *d*STORM data, it was clear from our imaging that the HDRs detected by *d*STORM were smaller and more heterogeneous than the segmented SSDs from our 3D-SIM data.

One of the motivations of this study is to compare data and analysis obtained from 3D-SIM imaging and dSTORM imaging. Ideally, we would be able to image the same synapses with both techniques to compare the resolved structures, however the technical and computational challenges of this type of correlative imaging (Reinhard et al., 2019) made these direct experiments unfeasible. Therefore, we created “pseudo-SIM” images by rendering our *d*STORM data to match the typical resolution achievable by SIM (Figure 2I; see section “Materials and Methods”). We then overlaid the NL2 or gephyrin *d*STORM coordinate map across the SIM-rendered image to compare HDR and SSD detection precision. By comparing these rendered images and coordinate maps we found that some, but certainly not all SSDs detected by SIM are composed of multiple smaller regions of heterogeneous density, that are only visible by higher resolution imaging techniques.

### Analysis of the Association Between Gephyrin and Neuroligin-2 at the Nanoscale Level

**FIGURE 3:**
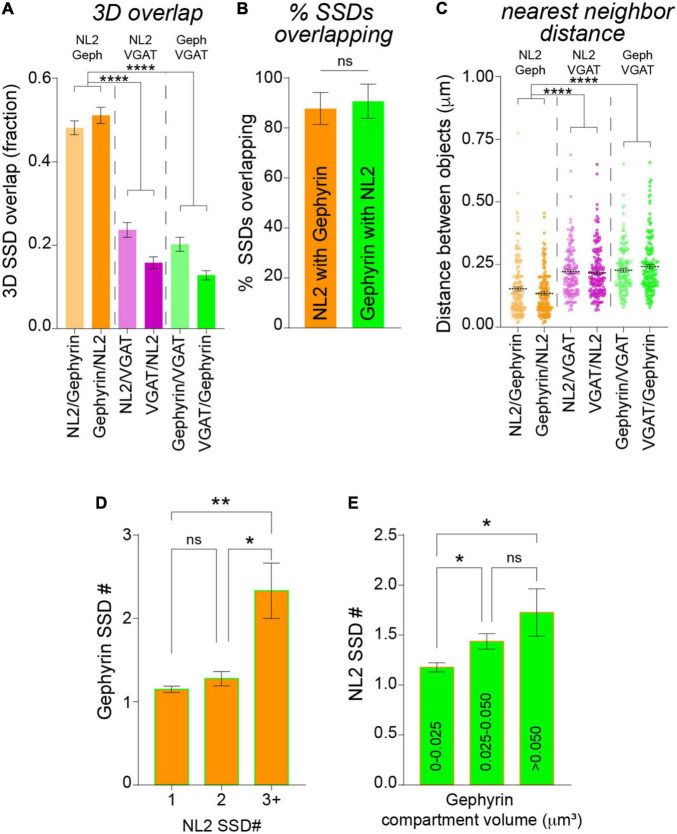
Analysis of NL2 and gephyrin SSD association by 3D-SIM. **(A)** Mean overlap fraction for NL2, gephyrin and VGAT SSDs. ****p < 0.0001 (Kruskal–Wallis, Dunn’s *post hoc*); *n* = 132 synapses. **(B)** Mean percentage of overlapping SSDs. *p* = 0.178 (Mann Whitney); *n* = 7 cells. **(C)** Center-to-center distances between neighboring NL2, gephyrin and VGAT SSDs. ****p < 0.0001 (Kruskal–Wallis, Dunn’s *post hoc*); *n* = 160–175 SSDs. Horizontal line denotes mean. **(D)** Mean gephyrin SSD number for NL2 compartments with 1, 2, or 3 SSDs per compartment. *p = 0.0225, **p = 0.0036; (Kruskal–Wallis, Dunn’s *post hoc*); *n* = 132 synapses. **(E)** Mean SSD number per NL2 compartment for a range of gephyrin compartment volumes. *p < 0.05 (Kruskal–Wallis, Dunn’s *post hoc*); *n* = 132 synapses.

Neuroligin-2 and gephyrin directly interact at inhibitory synapses (Poulopoulos et al., 2009). To assess whether NL2 nanoscale organization mirrors that of the gephyrin scaffold we asked whether NL2 and gephyrin SSDs are in close proximity to each other. NL2 and gephyrin SSDs were often observed paired together as shown in the example 3D-SIM images in Figure 2A. To assess whether this was a common feature for NL2 and gephyrin architecture across our 3D-SIM dataset, we used our segmentation work-flow to quantify the overlap and distance between neighboring SSDs within the 3D-SIM images. These data revealed a high degree of overlap between NL2 and gephyrin SSDs, compared with either NL2 or gephyrin SSDs with presynaptic VGAT substructures (Figure 3A). In agreement with this, ∼90% of NL2 SSDs overlapped gephyrin SSDs within an individual synapse (Figure 3B) indicating a high degree of association between these SSDs at the resolution of 3D-SIM. Measurement of the distance between NL2 SSDs and their nearest neighboring gephyrin SSDs showed that NL2 and gephyrin SSDs were significantly closer together than when compared to VGAT (Figure 3C). To give an indication of possible interdependence between NL2 and gephyrin SSD nanoscale organization we determined if the number of SSDs in the gephyrin compartment correlated with the number of SSDs in the NL2 compartment. NL2 compartments with larger numbers of SSDs were more likely to be associated with larger numbers of gephyrin SSDs (Figure 3D). Furthermore, larger gephyrin compartments correlated well with greater numbers of NL2 SSDs (Figure 3E), suggesting that larger post-synaptic domains have more NL2 distributed into SSDs. Together, analysis of our 3D-SIM data shows that at many synapses NL2 SSDs are close-to and overlap with gephyrin SSDs, suggesting that NL2 nanoscale organization may mirror that of the gephyrin scaffold.

**FIGURE 4:**
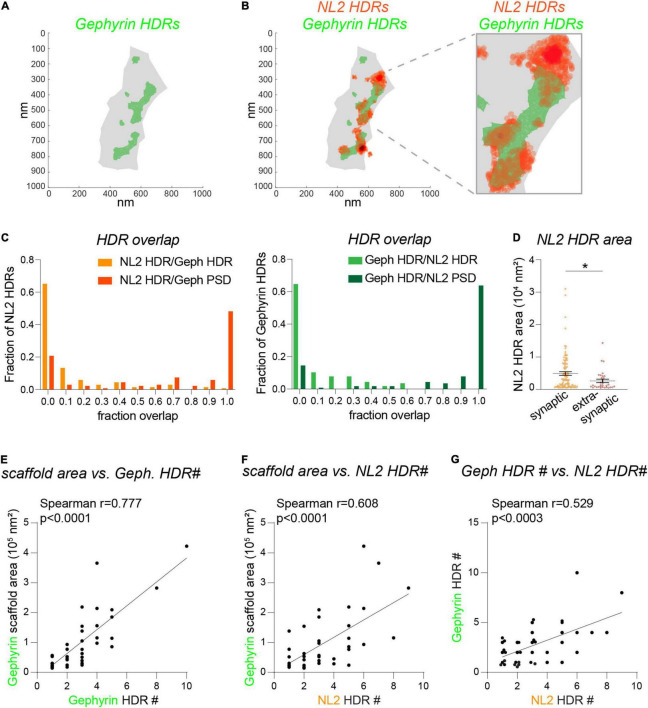
Fine detail of NL2 and gephyrin subsynaptic structure analyzed by *d*STORM. **(A)** *d*STORM plots showing gephyrin scaffold boundary (gray) and boundaries for gephyrin HDRs (green). **(B)** *d*STORM plots showing gephyrin boundaries (green) with NL2 HDR localizations overlaid (orange). **(C)** Distribution histograms showing the relative proportion of NL2 or gephyrin HDRs with different degrees of fraction overlap with HDRs (lighter colors) or scaffold regions (darker colors). **(D)** Area distribution for synaptic and extra-synaptic NL2 HDRs. HDRs are considered synaptic with a 0.23 fraction overlap or greater with the gephyrin scaffold. Horizontal line denotes mean. *p = 0.016 (Mann Whitney), *n* = 33–102 HDRs. **(E–G)** Correlation plots showing the liner relationship between the gephyrin scaffold area and the number of gephyrin HDRs **(E)** and the number of NL2 HDRs **(F)**. As a result, there is a corresponding correlation between the number of gephyrin HDRs and the number of NL2 HDRs **(G)**.

As discussed above, *d*STORM provides a far more detailed picture of nanoscale architecture for NL2 and gephyrin. To determine whether NL2 and gephyrin HDRs associate at the resolution level of *d*STORM, we quantified the area of overlap between HDRs based on the area fraction of each individual NL2 HDR overlapping with a gephyrin HDR (Figures 4A,B). As expected, the higher resolution of *d*STORM compared with 3D-SIM meant that NL2 and gephyrin HDRs displayed far less overlap compared with the SSDs identified by 3D-SIM. ∼ 65% of NL2 HDRs analyzed had ≤ 0.05 overlap with neighboring gephyrin HDRs (Figure 4C). However, in many cases NL2 HDRs were adjacent to the gephyrin HDR, with the peak of the NL2 localizations often lying next to the gephyrin HDR (as shown in Figure 4B). ∼75% of NL2 HDRs were positioned within the gephyrin scaffold region (the shaded gray area in Figures 4A,B), both in and outside of gephyrin HDRs. However, ∼25% of NL2 HDRs were found to be extra-synaptic. Quantification of NL2 HDR areas in and outside of the gephyrin scaffold region revealed that these extra-synaptic NL2 HDRs were significantly smaller than those inside the gephyrin scaffold (Figure 4D). Our 3D-SIM data suggest that larger gephyrin scaffolds contain more SSDs. To test whether this was also the case for the NL2 HDRs identified by *d*STORM we drew correlations between the number of HDRs and the area of the gephyrin scaffold (Figures 4E,F). There were strong correlations between the area of the scaffold and the number of either gephyrin or NL2 HDRs within the scaffold (Figures 4E,F), indicating that larger gephyrin scaffolds are likely able to support more NL2 HDRs. Moreover, the number of NL2 HDRs within a synapse correlated well with the number of gephyrin HDRs present (Figure 4G). Together, our *d*STORM analysis reveals substantially less overlap between NL2 and gephyrin HDRs than shown by 3D-SIM, with NL2 HDRs often found at the edge of neighboring gephyrin HDRs.

## Discussion

Multiple fluorescence-based imaging modalities have been applied to analyze the nanoscale architecture of synapses (Werner et al., 2021). These techniques increase resolution by different optical/computational methods, have their own set of advantages and disadvantages, and exhibit practical limits to the achievable resolution (Vangindertael et al., 2018). Here, we have used two orthogonal super-resolution imaging methods, 3D-SIM and *d*STORM, to study the subsynaptic distribution of the inhibitory synaptic adhesion protein NL2.

The two most notable differences between 3D-SIM and *d*STORM are the achievable resolution and data-type. The resolution of our 3D-SIM data is approximately half of what can be measured with standard confocal microscopy (∼120 nm laterally and ∼300 nm axially); our *d*STORM data is fit to a precision of 10–20 nm. 3D-SIM can discern distinct SSDs that are difficult/impossible to distinguish with confocal or wide-field imaging. In our previous work, we have shown that the organizational units at this resolution level (SIM-resolved SSDs) likely serve as “building blocks” of inhibitory synapses and emerge in, or are removed from, the synapses during different types of neuronal activity (Crosby et al., 2019; Garcia et al., 2021). We have found that NL2 is also organized into SSDs, of similar volume as gephyrin and GABA_*A*_R SSDs (Crosby et al., 2019), suggesting that NL2 SSDs could have a role in the nanoscale organization of GABA_*A*_Rs and gephyrin, and potentially the growth or shrinkage of the synapse.

With the more than five-fold improvement in resolution provided by *d*STORM, we found that the distribution of both gephyrin and NL2 localizations could be classified as either low-density or HDRs. HDRs were smaller than SIM-resolved SSDs; we postulate that SSDs are often made up of these mixed density regions, with one SSD perhaps containing multiple HDRs. Ideally, the same synapse would be imaged using each technique to confirm the smaller scale make-up of the SSDs, however, these types of experiments are technically challenging (Reinhard et al., 2019). Unsurprisingly, NL2 HDRs were more numerous and significantly smaller than NL2 SSDs identified by 3D-SIM. The higher resolution of *d*STORM also revealed reduced overlap between NL2 and gephyrin HDRs compared with that observed with 3D-SIM, with only about 25% of NL2 HDR showing an area overlap of 0.2 or greater with gephyrin HDRs. However, NL2 HDRs are often in close proximity to gephyrin HDRs and over 75% of NL2 HDR are found within the gephyrin post-synaptic scaffold. A notable consistency between our SIM and *d*STORM datasets is that the number of super-resolved substructures tended to scale with the overall size of the synapse: larger synapses generally harbored higher numbers of NL2 SSDs or HDRs. The same is true for gephyrin and GABA_*A*_R sub-structures, supporting the hypothesis that SSDs underly synapse growth and shrinkage (Crosby et al., 2019). It will be interesting to determine if NL2 SSDs emerge during plasticity protocols that induce overall growth of the synapse coupled with the addition of gephyrin and GABA_*A*_R SSDs. One critical distinction of our *d*STORM data is that, due in part to the single-molecule nature of the technique, we can detect smaller extrasynaptic clusters of NL2 that we have not observed using 3D-SIM. These extrasynaptic HDRs are smaller in area compared with their synaptic counterparts and could represent NL2 clusters that are trafficking to or from the synapse, or the seeding of a new synaptic site. Live super-resolution imaging such as live-cell PALM will be required to discern these possibilities.

In this study, our *d*STORM data has only been fit to provide 2D data; we therefore restricted our analysis to synapses that had a clear *en face* orientation. The implementation of 3D-STORM will improve our ability to interrogate inhibitory synapse substructure, in particular, the spatial relationship between pre- and post-synaptic elements. It is also important to note that these data represent static snap-shots of synaptic organization. We previously performed live-cell 3D-SIM experiments, imaging Gephyrin during homeostatic scaling (Crosby et al., 2019). During scaling-up of the synapse we observed the slow addition of SSDs with a corresponding growth of the gephyrin scaffold. In future studies using live-cell PALM, it will be interesting to determine the temporal stability of the HDRs we observe, and whether they increase in number during plasticity. It is possible that these regions morph and move over shorter time-scales while retaining the same overall mix of high and low-density within the boundaries of a more stable SSD.

## Data Availability Statement

The original contributions presented in the study are included in the article/Supplementary Material, further inquiries can be directed to the corresponding author.

## Ethics Statement

All animal work was reviewed and approved by the Institutional Animal Care and Use Committee at the University of Colorado.

## Author Contributions

KS and KC: conceptualization and methodology and writing – original draft. SG and MT: experiments. KC, SG, MT, SS, and KS: formal analysis and figure preparation. KS, KC, and SG: writing – review and editing. KS, MD, and MK: supervision and funding acquisition. All authors contributed to the article and approved the submitted version.

## Conflict of Interest

The authors declare that the research was conducted in the absence of any commercial or financial relationships that could be construed as a potential conflict of interest.

## Publisher’s Note

All claims expressed in this article are solely those of the authors and do not necessarily represent those of their affiliated organizations, or those of the publisher, the editors and the reviewers. Any product that may be evaluated in this article, or claim that may be made by its manufacturer, is not guaranteed or endorsed by the publisher.

## Footnotes

1
https://www.mathworks.com/matlabcentral/fileexchange?q=41093

2
http://bigwww.epfl.ch/sage/soft/mij/

3
github.com/DylanMuir/ReadImageJROI

4
https://github.com/VVvanL/NL2project_SmithLab
